# Prolyl hydroxylase 2 silencing enhances the paracrine effects of mesenchymal stem cells on necrotizing enterocolitis in an NF-κB-dependent mechanism

**DOI:** 10.1038/s41419-020-2378-3

**Published:** 2020-03-16

**Authors:** Hao Chen, Haifeng Zhang, Yue Zheng, Xiaohui Min, Yujun Luo, Weijie Zhou, Faxin Ma, Jinliang Li, Quan Lu, Chen Zhang, Huihua Cai, Weihong Sha

**Affiliations:** 1 Department of Gastroenterology, Guangdong Provincial People’s Hospital, Guangdong Academy of Medical Sciences, 510080 Guangzhou, China; 2 Department of Cardiology, Sun Yat-Sen Memorial Hospital, Sun Yat-Sen University, 510120 Guangzhou, China; 3 Department of Infectious Diseases, Sun Yat-Sen Memorial Hospital, Sun Yat-Sen University, 510120 Guangzhou, China; 4 Department of Obstetrics and Gynecology, Guangdong Provincial People’s Hospital, Guangdong Academy of Medical Sciences, 510080 Guangzhou, China

**Keywords:** Cell signalling, Mesenchymal stem cells

## Abstract

Treatment options for necrotizing enterocolitis (NEC) remain inadequate. Here we examined if and how prolyl hydroxylase 2 (PHD2) silencing enhances the paracrine effects of bone-marrow-derived mesenchymal stem cells (BM-MSCs) on NEC. In this study, BM-MSCs were transduced with lentiviruses containing GFP (GFP-MSC) or shPHD2-GFP constructs (PHDMSC), followed by intraperitoneal injection of the PHDMSC-conditioned medium (PHDMSC-CM) or the GFP-MSC-conditioned medium (MSC-CM) into a rat pup model of NEC. Our results showed that systemic infusion of PHDMSC-CM, but not MSC-CM, significantly improved intestinal damage and survival of NEC rats. Such benefits may involve the modulation of epithelial regeneration and inflammation, as indicated by the regeneration of intestinal epithelial/stem cells, the regulation of Treg cells function and pro-/anti-inflammatory cytokine balance. The mechanism for the superior paracrine efficacy of PHDMSC is related to a higher release of pivotal factor IGF-1 and TGF-β2. NF-κB activation was induced by PHD2 silencing to induce IGF-1 and TGF-β2 secretion via binding to IGF-1 and TGF-β2 gene promoter. Our work indicated that PHD2 silencing enhanced the paracrine effect of BM-MSCs on NEC via the NF-κB-dependent mechanism which may be a novel strategy for stem cell therapy on NEC.

## Introduction

Necrotizing enterocolitis (NEC) is the most common acute gastrointestinal illness in newborns, occurring in 7–10% of premature infants with a mortality rate of 20–50%^1^. The etiology and pathophysiology of NEC remain unclear, treatment options for infants affected by NEC are limited to supportive care, with twenty to forty percent of NEC cases progressing to surgical removal of the necrotic bowel^2^, and the survivors often need to deal with lifelong sequelae such as short bowel syndrome. Although NEC research has been conducted for decades, this disease remains the leading cause of morbidity and mortality in preterm infants^3^.

Stem cell therapy may be one of the most promising treatments for NEC. The abilities of stem cells to inhibit inflammation and tissue repair have aroused great interest for the treatment of NEC^4^. Stem cells have been reported to repair different types of small intestinal injuries^5–7^. The reparative effect of stem cells is not only manifested in stem cell migration and implantation into damaged tissues, but more importantly, stem cells also secrete a variety of trophic mediators, such as growth factors, immunomodulatory factors, and RNA-containing extracellular vesicles (EVs) to enhance tissue recovery^5–7^. Our recent study also shows that the paracrine mediators of mesenchymal stem cells collected by conditioned medium (MSC-CM) is beneficial for radiation induced intestinal injury (RIII)^8^.

Though MSC-CM therapy might be a viable alternative to stem cell transplantation, which is often hampered by low grafting efficiency and potential tumorigenesis, a major issue must be addressed before its clinical application is that the concentrations of trophic factors in CM are too low for therapeutic use^9^. Several reports including our previous study have shown that paracrine factors released by mesenchymal stem cells (MSCs) are insufficient for the repair of ischemic injury and/or small-intestinal mucosal injury^8,10^. One solution to deal with this problem is to modify the key genes of MSCs to enhance the secretion of trophic factors in MSCs. Modifications of AKT, vascular endothelial growth factor (VEGF), basic fibroblast growth factor (bFGF), and insulin-like growth factor (IGF-1) have been reported in previous studies to enhance the effect of MSCs paracrine secretions with limited efficacy^11^. Multiple clinical studies^12,13^ have also shown that a single prosurvival/angiogenic factor may not be sufficient to improve angiogenesis and repair tissue damage.

A new way to solve this problem is to regulate the upstream transcription factors that have many target genes. As an oxygen-dependent gene, prolyl hydroxylase 2 (PHD2) regulates two key transcription factors, hypoxia-inducible transcription factor-1α (HIF-1α), and nuclear factor-κB (NF-κB), which are involved in cell paracrine activity^14–16^. The activity of PHD2 decreases under hypoxia, which results in less hydroxylation and accumulation of HIF-1α and increased activity of NF-κB^17^. Previous studies have shown that PHD2 silencing enhances the secretion of a variety of bioactive factors, such as IGF-1 and angiopoietin-1^18^. Thus, PHD2 is an attractive candidate gene in gene therapy.

Considering the potential role of PHD2 on cell secretions, we used shRNA to silence PHD2 expression in MSCs (PHDMSC) and examined if and how PHD2 silencing enhances the paracrine effects of bone-marrow-derived mesenchymal stem cells (BM-MSCs) on NEC.

## Results

### Lentiviral transfection of shPHD2-GFP in BM-MSCs specifically knocked down the PHD2 expression

**Fig. 1:**
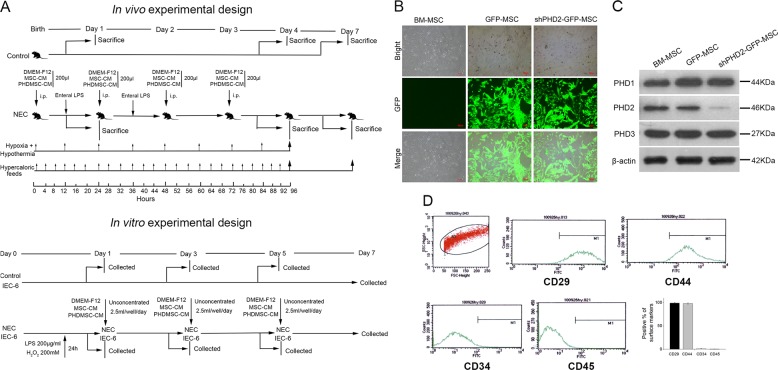
Lentiviral transduction of BM-MSCs with shPHD2-GFP specifically reduced PHD2 expression. **a** The experimental design is depicted for in vivo and in vitro experiments. For the in vivo experiments, newborn Sprague-Dawley rats delivered by caesarean section were not exposed (control) or exposed to experimental stresses (NEC), followed by a course of i.p. injection with control medium (DMEM-F12) or conditioned medium from GFP-transduced BM-MSCs (MSC-CM) or shPHD2-GFP-transduced BM-MSCs (PHDMSC-CM). Pups were either killed on days 1, 4, 7 for structural and functional examination or were monitored for survival status throughout the 7 day course of the experiment. For in vitro experiments, control rat intestinal epithelial IEC-6 cells or cells exposed to experimental stresses were cultured for 7 days. In parallel, IEC-6 cells were co-cultured in six-well plates with unconcentrated MSC-CM or PHDMSC-CM, and the proliferation and apoptosis were evaluated every other day as shown. **b** BM-MSCs with lentiviral transduction of BM-MSCs with GFP or shPHD2-GFP constructs. Scale bar = 100 μm. **c** Representative blots of PHD2, PHD1 and PHD3 expression in BM-MSCs with or without shPHD2. **d** Flow cytometric analysis shows that PHDMSC were positive for CD29, CD44 and negative for CD34, CD45, suggesting that the gene transfer procedure did not alter the MSCs phenotype.

This study established in vivo and in vitro experimental systems (Fig. 1a) to evaluate the therapeutic mechanisms of MSC-CM in NEC. The BM-MSCs were isolated and identified according to the methods described in our previous study^8^. Lentiviral vectors containing green fluorescent protein (GFP) or shPHD2-GFP were independently transfected into BM-MSCs (Fig. 1b). The shPHD2 specifically reduced the PHD2 protein expression without affecting PHD1 and PHD3 protein expression (Fig. 1c). The successfully transfected cells were obtained via fluorescence-activated cell sorting (FACS) enrichment and were further expanded in cell culture. These cultured cells were used for the preparation of the PHDMSC-derived conditioned medium (PHDMSC-CM).

To identify the phenotypes of BM-MSCs and PHDMSC, surface markers were identified by flow cytometry. Similar to BM-MSCs phenotype in our previous study^8^, flow cytometry demonstrated that PHDMSC were positive for stem cell markers CD29, CD44, but negative for haematopoietic lineage markers CD34, CD45 (Fig. 1d), suggesting that the gene transfer procedure did not alter the MSCs phenotype.

### PHDMSC-CM improved mortality and morbidity in rats with NEC by reducing structural and functional damage of the intestine

**Fig. 2:**
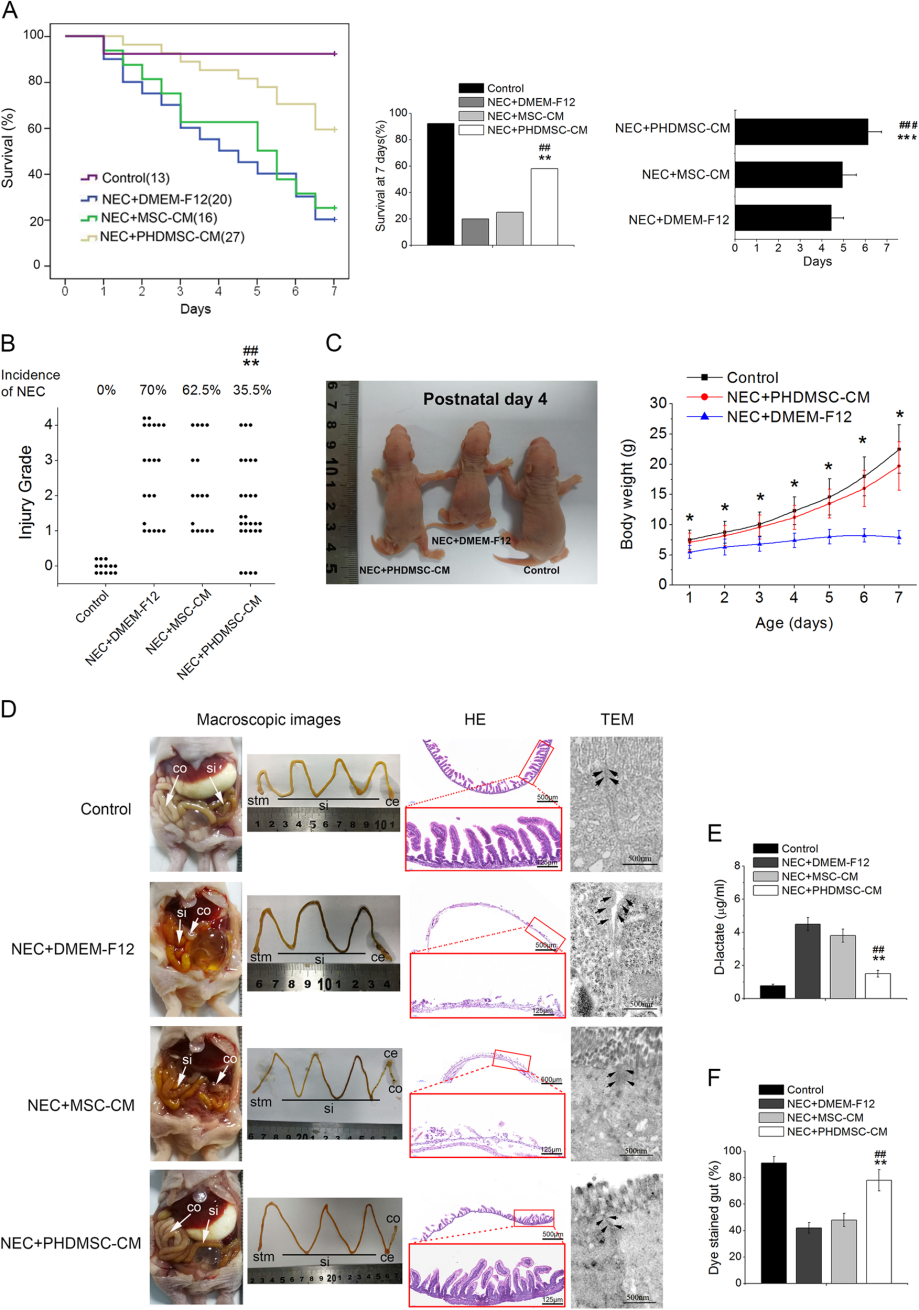
PHDMSC-CM improves mortality and morbidity in rats with NEC by reducing structural and functional damage of the intestine. **a** (Left panel) Cumulative survival for NEC rats infused with DMEM-F12, MSC-CM, or PHDMSC-CM was analyzed using the Kaplan–Meier method. The cumulated number of rats in each experimental group is presented in parenthesis. *P*-values were determined by log-rank testing. (Middle panel) During the first 7 days of life, NEC rats treated with PHDMSC-CM had a higher survival than those treated with DMEM-F12 or MSC-CM. (Right panel) Mean survival time. Data represent the mean ± SD. **P < 0.05 vs. NEC + DMEM-F12. ^##^*P* < 0.05 vs. NEC + MSC-CM. **b** Incidence and severity of NEC. Each dot represents an individual rat pup. **P < 0.05 vs. NEC + DMEM-F12. ^##^*P* < 0.05 vs. NEC + MSC-CM. **c** Macroscopic appearance 4 days after birth (Left panel), postnatal weight gain of pups 1–7 days after birth (Right panel). **d** Macroscopic, histologic and ultrastructural feature of the gastrointestinal tract on day 4 of life. (Left panel) Macroscopic appearance of the gastrointestinal tract. In NEC pups treated with DMEM-F12 and MSC-CM, dilation, significant hemorrhage, and discoloration were seen in the terminal ileum. (Middle panel) Images of H&E staining in the terminal ilea using light microscopy. (Right panel) The morphological changes of intestinal tight junction observed under transmission electron microscopy. Arrows, tight junctions; bars = 500 nm. **e** Intestinal permeability, measured as plasma level of D-lactate, was measured on day 4 of life in NEC. Values represent means ± SD of *n* = 3 animals, **P < 0.05 vs. NEC + DMEM-F12. ^##^*P* < 0.05 vs. NEC + MSC-CM. **f** Intestinal motility, measured with methylene blue dye migration, was measured at day 4 of life in NEC. Values represent means ± SD of *n* = 3 animals, **P < 0.05 vs. NEC + DMEM-F12. ^##^*P* < 0.05 vs. NEC + MSC-CM. NEC + DMEM-F12, NEC rats receiving DMEM-F12; NEC + MSC-CM, NEC rats receiving MSC-CM; NEC + PHDMSC-CM, NEC rats receiving PHDMSC-CM. stm stomach, si small intestine, ce cecum, co large intestine.

We first evaluated the effects of MSC-CM on the incidence and survival rates of NEC rat pups. Compared to NEC rats, the PHDMSC-CM treatment significantly improved the survival and reduced the incidence of NEC (Fig. 2a, b), and no significant difference on survival was observed between sexes (Supplementary Fig. 1). Although the mean survival time and the incidence of NEC in the MSC-CM treatment group were lower than those in the NEC injury group, no significant difference was found between these two groups (*P* > 0.05). Furthermore, the postnatal growth of NEC rats was severely impeded 7 days after birth. However, PHDMSC-CM treatment significantly improved postnatal growth retardation and postnatal weight gain of NEC rats (Fig. 2c).

We subsequently observed the effects of MSC-CM on small intestine epithelial structure and function. Control pups did not exhibit macroscopic damages, whereas NEC pups treated with DMEM-F12 and MSC-CM showed obvious injury in the distal ileum, with dilation, severe hemorrhage and discoloration. Ileal damages in rat pups with PHDMSC-CM treatment were significantly reduced compared to NEC rat pups (Fig. 2d). Hematoxylin and eosin (HE) staining showed complete villus destruction and transmural necrosis (Grade 3 or 4) in the NEC injury group and the MSC-CM treatment group on postnatal day 4. However, the intestinal histomorphology of the PHDMSC-CM group had significant improvement without showing any damage and necrosis (Grade 0, Fig. 2d). In the control group, the tight junctions (TJs) and desmosome were intact and clear between adjoining cells, and epithelial cell surface microvilli were arranged in neat rows. In the NEC group and MSC-CM group, TJ was open with the dotted crystal line structures obscured or disappeared in the TJ complex. Adherent epithelial cells became separated and the microvilli were damaged and sparse with irregular lengths and arrangements. However, the ultrastructural changes of the intestine in the PHDMSC-CM group were not measurably affected, which showed basically normal tight junction and regularly aligned microvilli, suggesting that the cell microstructure of the PHDMSC-CM group was significantly improved (Fig. 2d). The improvement of intestinal damage due to PHDMSC-CM was also reflected in gut function. Intestinal motility (methylene blue migration rate) and the intestinal permeability (measured as the plasma D-lactate concentration) of the NEC injury group were more severe than the normal controls, but they were significantly improved in PHDMSC-CM treated rats (Fig. 2e, f). Although the above indicators also appeared to improve after MSC-CM treatment compared to the NEC injury group, but no significant difference was found between the two groups (*P* > 0.05).

### PHDMSC-CM reduced apoptosis of the intestinal epithelial cells in vivo and in vitro

**Fig. 3:**
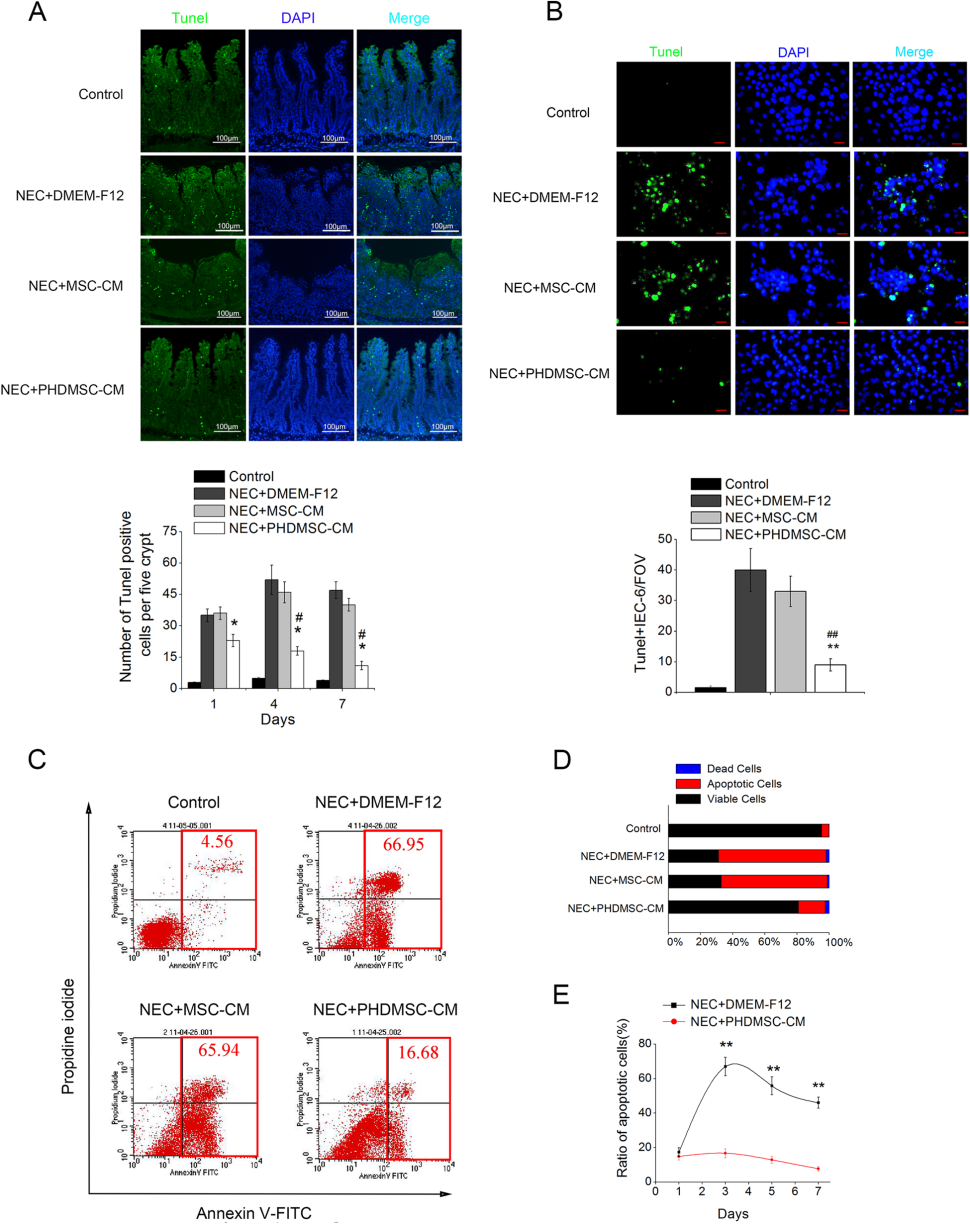
PHDMSC-CM reduces apoptosis of intestinal epithelial cells in rats with NEC in vivo and in vitro. **a** Apoptosis was assayed by TUNEL staining on day 4 of life (upper panel). Scale bars 100 μm. (lower panel) Quantification of TUNEL-positive cells. *n* = 3 in each group. The number of positive cells in 5 crypts was scored in 100 crypts per section and reported as mean ± SD. *P < 0.05 vs. NEC + DMEM-F12. ^#^*P* < 0.05 vs. NEC + MSC-CM. **b** Apoptosis of IEC-6 was assayed by TUNEL staining 3 days after exposure (upper panel). Scale bars 25 μm. (lower panel) Quantification of TUNEL-positive IEC-6 cells. Data are reported as means ± SD for 10 random fields per wells from four replicate wells per group. **P < 0.05 vs. NEC + DMEM-F12. ^##^*P* < 0.05 vs. NEC + MSC-CM. FOV, field of view. **c** Apoptosis and cell death of IEC-6 were evaluated quantitatively by flow cytometry after PI/Annexin V staining. The right lower quadrant (RLQ) contains early apoptotic cells, and the upper right quadrant (RUQ) contains late apoptotic and necrotic cells. **d** The percentage of total apoptotic cells and dead cells under each condition from panel C are shown. **e** The ratio of apoptosis IEC-6 cells was determined by PI/Annexin V staining 1, 3, 5, and 7 days after exposure. Data represent means ± SD of three independent experiments. **P < 0.05 vs. NEC + DMEM-F12.

The number of TUNEL-positive intestinal nuclei in the small intestine tissue sections was used as the evaluation criterion for the degree of apoptosis in this study. Compared to the normal control rat pups, the number of TUNEL-positive cells from NEC pups was ~10-fold higher 4 days after NEC injury, especially in the intestinal crypts (Fig. 3a), suggesting that the intestinal stem cells (ISCs) in the intestinal crypts were severely damaged. This finding was consistent with a report by Nino et al.^19^ showing that NEC is characterized by apoptosis of intestinal stem cells located at the bottom of the intestinal crypts. Compared to the NEC injury group, the degree of apoptosis in the small intestine epithelium 1, 4, and 7 days after the PHDMSC-CM treatment was significantly reduced in the NEC model. Although apoptosis also tended to be reduced in the MSC-CM group, no statistical significance was found.

To rule out any inhibitory effect of PHDMSC-CM on apoptosis that may occur by indirectly affecting the immune system, we determined the effect of PHDMSC-CM in vitro on LPS/H2O2 treated IEC-6 cells. Co-culturing with PHDMSC-CM, but not with MSC-CM, protected LPS/H2O2 treated IEC-6 cells against apoptosis as assessed by TUNEL staining (Fig. 3b). Consistent with this result, Annexin V and PI double staining also revealed that co-culture with PHDMSC-CM dramatically reduced the apoptosis of LPS/H2O2 treated IEC-6 cells compared to those cultured with DMEM-F12 and MSC-CM on day 3, day 5 and day 7 (Fig. 3c–e).

### PHDMSC-CM promoted intestinal epithelial cell proliferation in vivo and in vitro

**Fig. 4:**
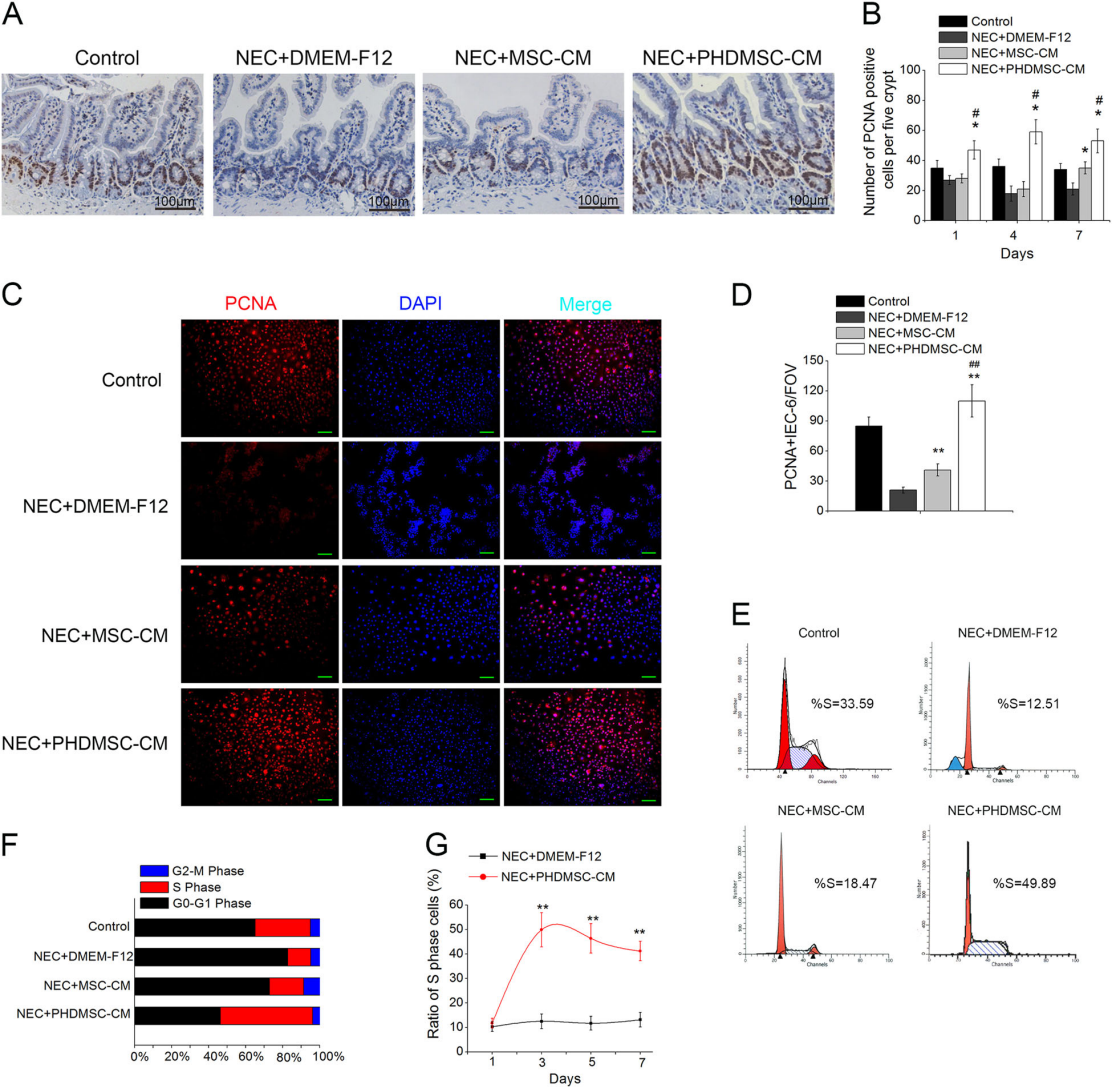
PHDMSC-CM increases the proliferation of intestinal epithelial cells in rats with NEC in vivo and in vitro. **a** The proliferation of intestinal epithelial cells was examined by immunohistochemical staining with proliferating cell nuclear antigen (PCNA). Intestinal tissue samples were collected and analyzed on day 4 of life. Scale bars 100 μm. **b** Quantification of PCNA-positive cells. *n* = 3 in each group. The number of positive cells in 5 crypts was scored in 100 crypts per section and reported as mean ± SD. *P < 0.05 vs. NEC + DMEM-F12. ^#^*P* < 0.05 vs. NEC + MSC-CM. **c** Immunohistochemical staining of IEC-6 cells with PCNA 3 days after exposure. Scale bars 50 μm. **d** Quantification of PCNA-positive cells for the treatment groups in panel **c**. Data are reported as mean ± SD for 10 random fields per wells from four replicate wells per group. **P < 0.05 vs. NEC + DMEM-F12. ^##^*P* < 0.05 vs. NEC + MSC-CM. FOV field of view. **e** The cell cycle status were detected by flow cytometry based on DNA content. **f** The percentage of S phase cells and G1 phase cells for each culture condition was determined. **g** The ratio of S phase cells was examined 1, 3, 5, and 7 days after exposure to experimental stresses. Data represent mean ± SD of three independent experiments. **P < 0.05 vs. NEC + DMEM-F12.

Proliferating cell nuclear antigen (PCNA)-positive intestinal cells in the small intestine tissue sections were used to evaluate the proliferating cells. The numbers of PCNA-positive cells in the PHDMSC-CM group on postnatal days 1, 4, and 7 were significantly higher than those in the MSC-CM group and the NEC injury group (Fig. 4a, b), and were especially obvious in the intestinal crypts. Similar to the in vivo observation, the number of PCNA-positive cells was significantly increased in the LPS/H2O2 treated IEC-6 co-cultured with PHDMSC-CM (Fig. 4c, d). In addition, starting from the third day of co-culture, PHDMSC-CM rather than MSC-CM co-culture significantly increased the proportion of S phase cells in the IEC-6 cell cycle (Fig. 4e–g).

### PHDMSC-CM promoted intestinal epithelial stem cell regeneration in NEC

**Fig. 5:**
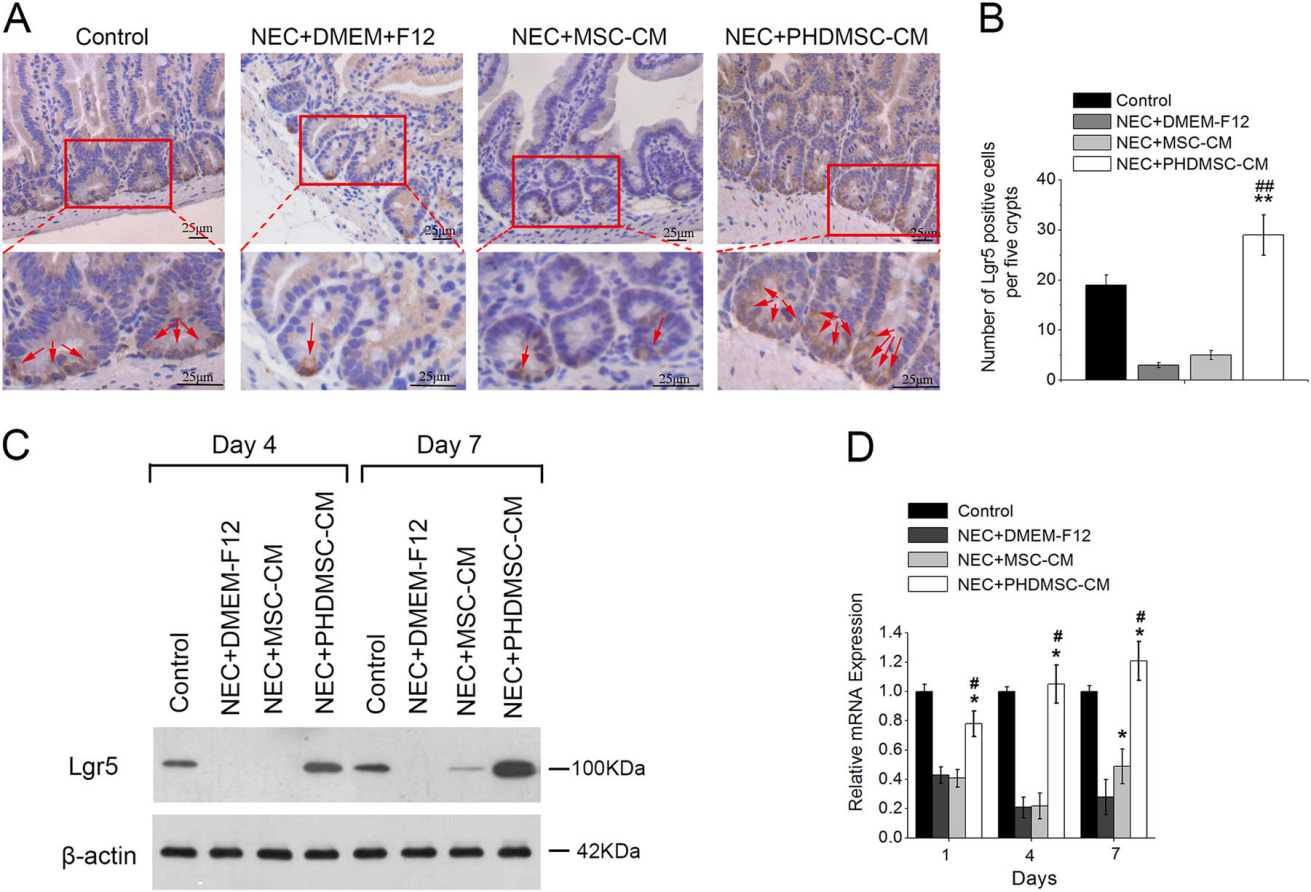
PHDMSC-CM promotes regeneration of intestinal stem cells in rats with NEC. **a** Immunohistochemical staining with Lgr5. Intestinal tissue samples were collected and analyzed on day 4 of life. Scale bars 25 μm. **b** Quantification of Lgr5-positive cells. *n* = 3 in each group. The number of positive cells in 5 crypts was scored in 100 crypts per section and reported as mean ± SD. **P < 0.05 vs. NEC + DMEM-F12. ^##^*P* < 0.05 vs. NEC + MSC-CM. **c** The protein levels of Lgr5 in the ileum mucosa of rats were detected by Western blot assays on days 4 and 7 of life with β-actin as the internal control. **d** *Lgr5* mRNA expression in the ileum mucosa of rats after exposure was evaluated by quantitative real-time RT-PCR. *β-actin* was used as a loading control. Values represent means ± SD; *n* = 3 in each group. *P < 0.05 vs. NEC + DMEM-F12. ^#^*P* < 0.05 vs. NEC + MSC-CM.

We further evaluated the effect of PHDMSC-CM on Lgr5^+^ (Leucine-rich repeat-containing G-protein coupled receptor 5) intestinal stem cells (ISCs), the key cells for intestinal epithelial cell regeneration in NEC. Lgr5-expressing cells are actively proliferating ISCs responsible for the maintenance of the intestinal epithelium. The loss of Lgr5^+^ ISC pools has been implicated in NEC pathophysiology as it can cause decreased capacity for intestinal epithelial tissue regeneration and renewal that is observed in NEC^19^. In this study, expression of the Lgr5^+^ ISCs was very scattered 3 days after the NEC injury, with only 1-2 Lgr5-positive cells located at the very bottom of the intestinal crypt. In contrast, after the PHDMSC-CM treatment, the number of Lgr5-positive cells was significantly increased (Fig. 5a, b). These results were confirmed by western blot analysis (Fig. 5c) and real-time quantitative PCR (Fig. 5d), showing dramatic differences on postnatal days 1, 4, and 7. These results were consistent with our previous findings that PHDMSC-CM inhibited apoptosis and promoted cell proliferation in the intestinal crypts, suggesting that PHDMSC-CM possibly accelerated the regeneration of small intestine epithelial cells by inhibiting Lgr5^+^ ISCs apoptosis and promoting the Lgr5^+^ ISCs proliferation.

### PHDMSC-CM downregulated the inflammatory response in NEC at systemic and mucosal levels

**Fig. 6:**
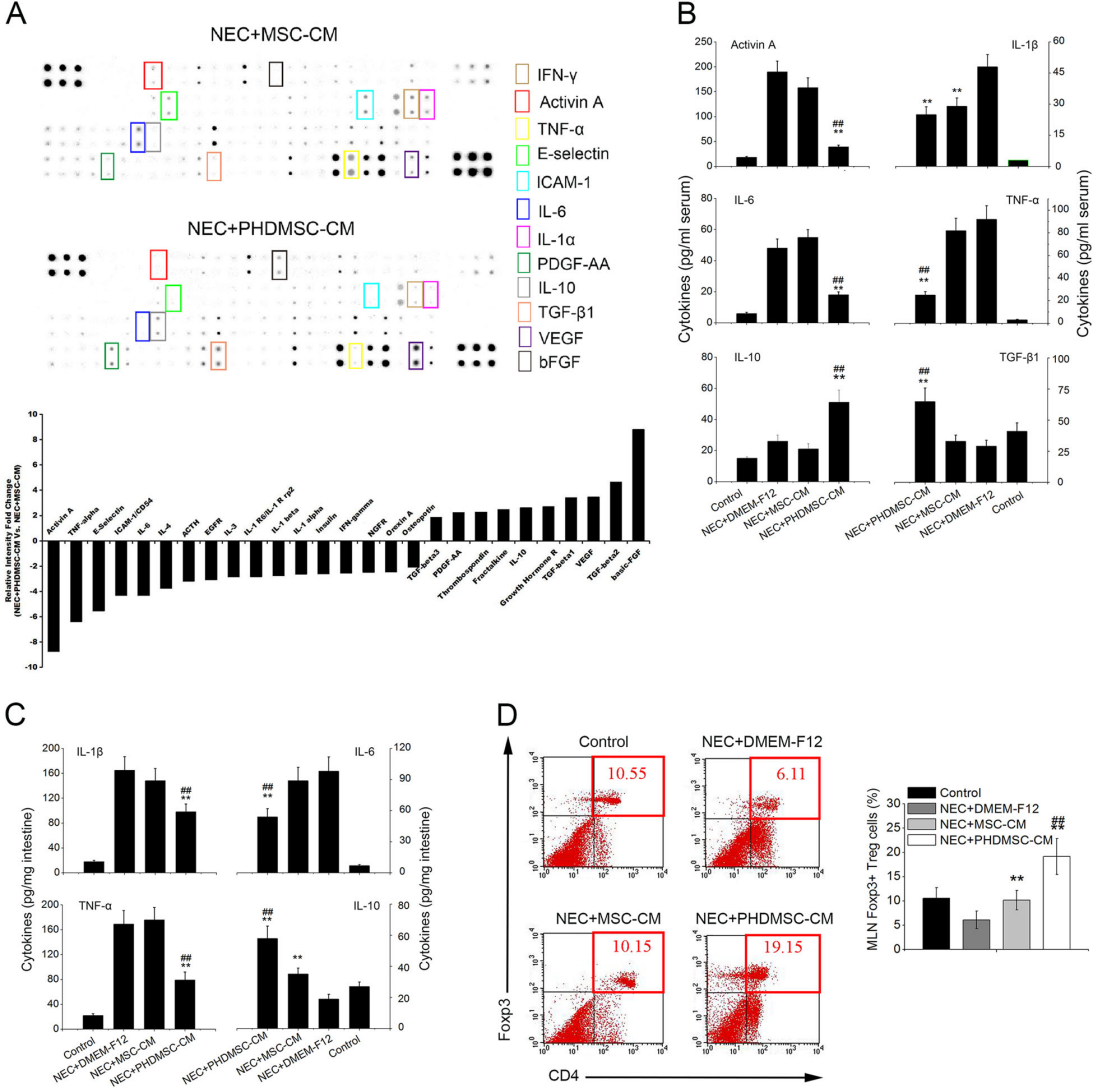
PHDMSC-CM down-regulates inflammatory responses in rats with NEC at systemic and musocal level. **a** (Upper panel) Representative images of the cytokine antibody array are shown. The rectangles highlight cytokines significantly different between NEC rats receiving PHDMSC-CM and NEC rats receiving MSC-CM (fold change > 2.0). Each sample consisted of a pool of serum from five different donor rats. Each measurement was duplicated reproducibly. (Lower panel) The graphs show the relative intensity fold changes of the 27 cytokines detected by antibody-based protein array. **b** The same serum samples were assayed by ELISA for six selected cytokines. **P < 0.05 vs. NEC + DMEM-F12. ^##^*P* < 0.05 vs. NEC + MSC-CM. **c** The levels of pro/anti-inflammatory cytokines in intestinal protein extracts on day 4 of life were determined by ELISA. *n* = 4 rats/group. **P < 0.05 vs. NEC + DMEM-F12. ^##^*P* < 0.05 vs. NEC + MSC-CM. **d** The percentages of CD4^+^Foxp3^+^ Treg cells in the CD4^+^ population of mesenteric lymph nodes (MLNs) were determined by flow cytometry (4–5 rats/group). Tissue samples were collected on day 4 of life. **P < 0.05 vs. NEC + DMEM-F12. ^##^*P* < 0.05 vs. NEC + MSC-CM.

To investigate the immunomodulatory effects of MSC-CM on inflammation in NEC, we first used antibody microarrays to monitor 90 factors simultaneously and to evaluate serum inflammatory cytokines in NEC rats treated with MSC-CM or PHDMSC-CM. Compared to the MSC-CM treatment group, 10 serum cytokines, which included bFGF, VEGF, platelet-derived growth factor (PDGF)-AA, transforming growth factor beta 1 (TGF-β1), interleukin (IL)-10, and several others, were significantly increased (>2-fold) in the PHDMSC-CM treatment group. Additionally, 17 cytokines, including activin A, intercellular adhesion molecule 1 (ICAM-1), E-selectin, IL-1β, IL-4, IL-6, tumor necrosis factor alpha (TNF-α), interferon-gamma (IFN-γ), and several others, were significantly reduced (Fig. 6a). We further selected 6 factors closely related to inflammation for ELISA. Compared to the MSC-CM, PHDMSC-CM significantly reduced serum activin A, IL-1β, IL-6, and TNF-α expression in the NEC rat pups and enhanced serum TGF-β1 and IL-10 concentrations, thus showing a systemic anti-inflammatory effect (Fig. 6b). Similar to the systemic anti-inflammatory effect observed in the serum, PHDMSC-CM but not MSC-CM treatment significantly reduced proinflammatory cytokines and increased anti-inflammatory cytokines in the small intestinal mucosa (Fig. 6c). Down-regulation of inflammation and the elevated IL-10 levels prompted us further to investigate the effect of PHDMSC-CM on Treg cells. As shown in Fig. 6c, PHDMSC-CM treatment significantly increased the number of CD4^+^Foxp3^+^ Treg cells in the mesenteric lymph node (MLN) of the NEC rat pups compared to those in NEC rats and PHDMSC-CM treated rats. (Fig. 6d).

### PHD2 silencing induced changes in the secretome of BM-MSCs

**Fig. 7:**
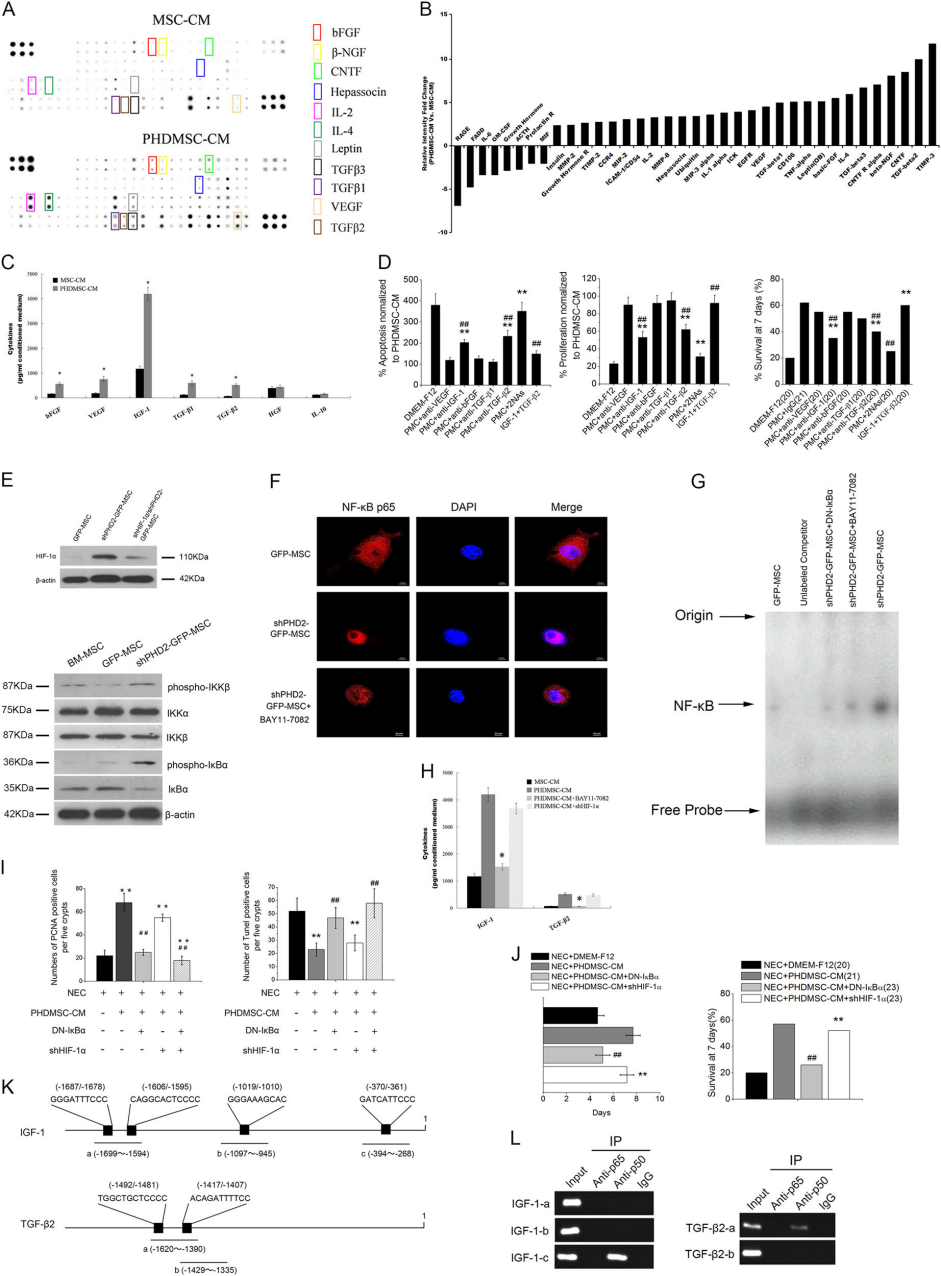
PHD2 silencing enhances BM-MSCs paracrine effect on NEC via the NF-κB pathway. **a** Identification of cytokines of PHDMSC-CM that are potentially beneficial to intestinal recovery. Representative images of the cytokine antibody array are shown. The rectangles highlight cytokines higher in PHDMSC-CM than MSC-CM (fold change > 2.0). Each sample consisted of a pool of unconcentrated conditioned medium from five different donor cells which were derived and sampled independently. Each measurement was duplicated reproducibly. **b** The graphs show the relative intensity fold changes of the 36 cytokines detected by antibody-based protein array. **c** Unconcentrated conditioned medium was assayed by ELISA for six selected cytokines. *N* = 6 independent samples. *P < 0.05 vs. MSC-CM. **d** The effect of neutralization of selected cytokines or addition of selected exogenous cytokines on apoptosis (Left panel) and proliferation (Middle panel) of IEC-6. The apoptosis and proliferation were evaluated by quantification of TUNEL-positive and PCNA-positive IEC-6 cells in vitro 3 days after exposure, respectively. *N* = 6. **P < 0.05 vs. NEC + PHDMSC-CM. ^##^*P* < 0.05 vs. NEC + DMEM-F12. (Right panel) The effect of neutralization of selected cytokines or addition of selected exogenous cytokines on survival of NEC rat pups during the first 7 days of life. The cumulated number of rats in each experimental group is presented in parenthesis. **P < 0.05 vs. NEC + DMEM-F12. ^##^*P* < 0.05 vs. NEC + PHDMSC-CM + IgG. **e** (Upper panel) Representative blots of Western blotting analysis of HIF-1α protein expression in BM-MSCs with or without PHD2 silencing. (Lower panel) Representative blots for phospho-IKKΒ, total IKKα, total IKKΒ, phospho-IκBα, and total IκBα in BM-MSCs with or without PHD2 silencing. β-actin is used as control. **f** Effect of PHD2 silencing on nuclear translocation of NF-κB in BM-MSCs. Scale bars 10 μm. **g** The DNA-binding activities of NF-κB in BM-MSCs with or without PHD2 silencing were estimated by electrophoretic mobility shift assay (EMSA). The specificity of the DNA/protein was determined by competition reactions in which a 100-fold molar excess of unlabeled NF-κB oligonucleotide (specific competitor). **h** The effect of HIF-1α silencing and NF-κB inhibition on pivotal cytokines secretion of BM-MSCs were determined by ELISA. *N* = 6 independent samples. *P < 0.05 vs. PHDMSC-CM. **i** The effect of NF-κB inhibition on BM-MSC paracrine mediated effect on intestinal epithelial cells in rats with NEC. NF-κB inhibition but not HIF-1α silencing blocked the therapeutic potential of PHDMSC-CM to ameliorate the proliferation (left panel) and apoptosis (right panel) of intestinal epithelial cells in rats with NEC which were evaluated by quantification of TUNEL-positive and PCNA-positive cells in histological sections, respectively. **P < 0.05 vs. NEC + DMEM-F12. ^##^*P* < 0.05 vs. NEC + PHDMSC-CM. **j** Improved survival of rats with NEC receiving PHDMSC-CM was reversed by NF-κB inhibition. The cumulated number of rats in each experimental group is presented in parenthesis. **P < 0.05 vs. NEC + DMEM-F12. ^##^*P* < 0.05 vs. NEC + PHDMSC-CM. **k** Schematic diagram of primer pairs in ChIP analysis. (Upper panel) The rIGF-1 promoter region contains four NF-κB-binding sites. Three primer pairs were designed to detect regions a, b, and c in ChIP assays. (Lower panel) The rTGF-β2 promoter region contains two NF-κB-binding sites. Two primer pairs were designed to detect regions a and b in ChIP assays. **l** The p50 bound to the κB site within the promoter of rIGF-1 and rTGF-β2 detected by ChIP assay. ChIP analysis was performed using anti-p65, anti-p50, or control IgG antibodies for immunoprecipitation followed by PCR using primers for the specific rIGF-1/rTGF-β2 promoter region in which the predicted NF-κB site is located. The PCR products were subjected to electrophoresis on 2% agarose gel. These results are representative of three independent experiments.

To investigate the possible reasons for these differential effects, we first monitored the changes in the secretome of BM-MSCs with and without PHD2 silencing using a protein microarray to analyze 90 rat cytokine changes simultaneously in the MSC-CM and the PHDMSC-CM. Compared to MSC-CM, 27 cytokines were significantly increased (>2-fold) in PHDMSC-CM (Fig. 7a, b). These cytokines could be classified into three categories according to their functions: the first category contained growth factors which promoted regeneration, including bFGF, VEGF, beta-nerve growth factor (β-NGF), hepassocin, and ciliary neurotrophic factor (CNTF); the second category included chemokines and matrix metalloproteinases, such as C–C chemokine receptor type 4 (CCR4), chemokine (C–X–C motif) ligand 2 (CXCL-2), chemokine (C–C motif) ligand 20 (CCL-20), tissue inhibitor of metalloproteinases (TIMP)-2/3, and matrix metalloproteinase (MMP)-2/8, which can enhance MSCs or immune cell trafficking to a certain location; and the third category contained immunomodulatory factors, such as TGF-β1/2/3.

Based on the previous findings^20,21^ that IGF-1, HGF (IGF-1 and HGF were not included in the 90 target cytokines in the cytokine microarray) promote regeneration of epithelial and ISCs in the intestine. This study subsequently used ELISA to further confirm the levels of IL-10, IGF-1, and HGF in PHDMSC-CM together with 4 other growth factors (bFGF, VEGF, TGF-β1, and TGF-β2) screened upregulated in PHDMSC-CM. In PHDMSC-CM, the concentrations of the bFGF, VEGF, IGF-1, TGF-β1, and TGF-β2 were 556.91 ± 72.85, 753.53 ± 109.47, 4195.47 ± 159.57, 596.52 ± 89.32, and 512.76 ± 45.91 pg/ml, respectively. These concentrations were 3–10 folds higher than when MSC-CM was used (Fig. 7c). Although results of ELISA also confirmed the presence of HGF and IL-10 in PHDMSC-CM, no significant difference in their levels was observed between PHDMSC-CM and MSC-CM.

### IGF-1 and TGF-β2 plays a critical role in PHDMSC-CM mediated NEC recovery

Since VEGF, bFGF, IGF-1, TGF-β1, and TGF-β2 were significantly elevated after PHD2 silencing, and previous studies had suggested that these cytokines played an active role in NEC repair^20,22,23^, we further studied if the beneficial effect of PHDMSC-CM on NEC was associated with these cytokines. When addition of individual neutralizing antibodies for each of these cytokines, the effect of PHDMSC-CM on apoptosis and proliferation of IEC-6 was partially inhibited by antibodies against IGF-1 or TGF-β2 individually and further blocked by combined neutralization of IGF-1 and TGF-β2. Similarly, the survival effect of PHDMSC-CM on NEC pups was blocked by antibodies against IGF-1 + TGF-β2 from 61.2% to 25%. In addition, combined infusion of IGF-1 and TGF-β2 showed similar protective effect on intestinal proliferation/apoptosis and survival rates compared to PHDMSC-CM (Fig. 7d). Moreover, we further observed increased expressions of IGF-1 and TGF-β2 in NEC tissue after PHDMSC-CM treatment (Supplementary Fig. 2), which suggests a pivotal role of IGF-1 and TGF-β2 in PHDMSC-CM mediated NEC recovery.

### Paracrine protective effects of BM-MSCs with PHD2 silencing depended on the NF-κB pathway

Previous studies have shown that PHD2 affects the secretions of MSCs through HIF-1α and NF-κB pathways^14,24^. To clarify the mechanism that mediates the difference in the paracrine profiles of MSCs with and without PHD2 silencing, we first observed the changes in these two pathways before and after silencing the PHD2. PHD2 silencing significantly enhanced the expression of HIF-1α, which can be interrupted by shHIF-1α (Fig. 7e). PHD2 silencing increased the phosphorylation of IKKβ, and further increased the phosphorylation and degradation of IκBα (Fig. 7e) thereby enhancing nuclear translocation (Fig. 7f) and DNA binding activity of NF-κB (Fig. 7g), which can be interrupted by the NF-κB inhibitor BAY11-7082 or a dominant-negative mutant of IκBα (DN-IκBα). BAY11-7082 blocked the elevation of pivotal cytokines (IGF-1 and TGF-β2) induced by PHD2 silencing (Fig. 7h). In addition, DN-IκBα blocked the beneficial effects of PHDMSC-CM on small intestine epithelial cell proliferation (PCNA-positive cell number), apoptosis (TUNEL-positive cell number), and survival of NEC rats (Fig. 7i, j). Although we also observed that shHIF-1α dramatically attenuated VEGF elevation induced by PHD2 silence (data not show), shHIF-1α did not attenuate the protective effect of PHDMSC-CM on NEC. Moreover, CM from lipopolysaccharide (LPS, an NF-κB stimulator)-treated BM-MSCs provides beneficial effects on apoptosis and proliferation of LPS/H2O2 treated IEC-6 cell (Supplementary Fig. 3). This suggested that the benefit paracrine effect of BM-MSCs with PHD2 silencing on NEC was NF-κB dependent.

We next explored the molecular basis of NF-κB mediated secretion of pivotal factors. We demonstrated that the IGF-1 and TGF-β2 mRNA was significantly increased by NF-κB stimulator LPS (Supplementary Fig. 4). To further confirm the binding of NF-κB to the promoter of IGF-1 and TGF-β2, chromatin immunoprecipitation (ChIP) analysis was performed. Four NF-κB binding sites in IGF-1 promoter and two NF-κB binding sites in TGF-β2 promoter (Fig. 7k) were predicted with program ALGGEN-PROMO (http://alggen.lsi.upc.es/cgi-bin/promo_v3/promo/promoinit.cgi?dirDB=TF_8.3). A ChIP assay showed that the predicted site of the IGF-1 and TGF-β2 promoter binded to NF-κB p50 (Fig. 7l). These results indicate that NF-κB activation may directly regulate the transcription and secretion of IGF-1 and TGF-β2 via binding to IGF-1 and TGF-β2 gene promoter.

## Discussion

The present study confirmed our hypothesis that PHD2 silencing enhances the paracrine efficacy of BM-MSCs on NEC, and demonstrated that PHDMSC-CM, but not MSC-CM, (1) improves survival and promotes the structural and functional restoration of NEC by regulating epithelial regeneration and inflammatory response; (2) activates NF-κB to increase their secretion of pivotal factor IGF-1 and TGF-β2 via binding to IGF-1 and TGF-β2 gene promoter, therefore provides superior therapeutic efficacy on NEC.

There are several important findings in our work. The first was that we showed the role and mechanism of MSC-CM on NEC. Thus far, use of MSC-CM as treatment for NEC has been rarely reported, and only one study has shown that conditioned medium from amniotic fluid stem cells (AFS) may have similar reparative effects to AFS on NEC^25^. In this study, we report that PHDMSC-CM, but not MSC-CM, can improve NEC injury by regulating cellular homeostasis via effects on the apoptosis and proliferation of intestinal epithelium/stem cells, and skewing the inflammatory environment towards an anti-inflammatory profile partially due to their immune modulation of Treg cells, suggesting that PHDMSC-CM has the comparable capability to MSCs transplantation^26^. Therapeutic differences in NEC between conventional MSC-CM and AFS-CM may be due to the differences of their paracrine profiles. A previous study^27^ has shown that AFS releases a number of growth factors at concentrations higher than those of BM-MSCs, thereby more substantially promoting the regeneration and maturation of skin blood vessels.

Second, to explain the therapeutic difference in NEC between PHDMSC-CM and MSC-CM, we monitored the changes in BM-MSCs paracrine secretions after silencing PHD2. By using antibody microarrays and neutralizing antibodies, IGF-1 and TGF-β2 were identified as the pivotal mediators in PHDMSC-CM-mediated NEC recovery. Combined infusion of these 2 pivotal mediators can almost replicate the protective effect of PHDMSC-CM on NEC, suggesting a new strategy for clinical application on NEC. The observation that IGF-1 and TGF-β2 are required for PHDMSC-CM-mediated recovery in NEC is consistent with previous observations that IGF-1 and TGF-β2 may play critical roles in NEC. For example, IGF-I increases intestinal protein contents, enterocyte migration rates^28^ and proximal small intestinal enzyme activity in newborn rats^29^ whereas prolonged low serum concentrations of IGF-I in premature infants are associated with the development of NEC^30^. Animal studies, using a murine model of NEC, indicated that exogenous IGF-1 administration resulted in improved survival and incidence rate of NEC, via effects on the apoptosis and inflammatory responses of intestinal epithelium cells^20^. TGF-β2, an important anti-inflammatory protein in milk and colostrum, contains in high quantities in human breast milk^31^ whereas deficiency of TGF-β2 was observed in the intestine of formula-fed premature infants and was even more pronounced in premature infants with NEC^32–34^. It has been reported that TGF-β2 suppresses macrophage cytokine expression and mucosal inflammatory responses in the developing human intestine, and shown using murine models that enterally administered TGF-β2 can protect against NEC^34^, supporting our observation that combination treatment of IGF-I and TGF-β2 promotes NEC recovery.

Lastly, we explored the specific mechanisms involved in the enhanced paracrine potential of PHDMSC. PHD2 has been considered a key oxygen sensor and primary HIF prolyl hydroxylase in cells^14^. Multiple studies^35,36^ have shown that PHD2 silencing enhanced the survival of transplanted stem cells and the secretion of paracrine factors by increasing the stability of HIF-1α, thereby repairing tissue damage. In present study, although we observed that shPHD2 enhanced the VEGF secretion of BM-MSCs through HIF-1α, HIF-1α silencing did not weaken the protective effects of PHDMSC-CM on NEC, indicating that their paracrine effects are independent of HIF-1α. The NF-κB pathway is an important pathway for PHD2 to promote angiogenesis^37^. Activation of the NF-κB pathway does not only enhance the viability of MSCs but also promotes the secretion of various paracrine factors^16^. Our study showed that PHD2 silencing induces NF-κB activation probably by enhancing IKKβ activity and thus reducing IκBα, whereas blocking of the NF-κB pathway not only significantly suppressed the secretion of pivotal factor but also reversed the protective effects of PHDMSC-CM in NEC. ChIP assays further indicate that NF-κB p50 can bind to IGF-1 and TGF-β2 promoter. Together, our data indicates that activation of NF-κB induced by PHD2 silencing can directly induce IGF-1 and TGF-β2 secretion via binding to IGF-1 and TGF-β2 gene promoter.

PHD2 is a gene extensively expressed in various types of cells. PHD2 silencing enhances the secretion of paracrine factors in fibroblasts^38^, and cardiomyocytes^39^, thereby promoting tissue repair. Therefore, PHD2 silencing did not only significantly enhance the paracrine capacity of BM-MSCs but also enhanced the paracrine capacity of other cells. Although PHD2 has broad prospects in gene therapy, its safety may limit its further application in clinical practice. Multiple studies have shown that down-regulation of PHD2 promotes tumor angiogenesis and might thus promote tumorigenesis and tumor metastasis^40,41^. Although other reports confirmed that downregulation of PHD2 resulted in no evidence of tumorigenesis in a mouse model for 1.5 years^42^, the possibility of tumorigenesis caused by the inhibition of PHD2 can not be ruled out. In present study, we use PHD2-modified stem cells in vitro to provide protective paracrine effects on NEC without the aforementioned safety issues, suggesting a feasible choice for NEC treatment and even for other types of tissue damage.

In conclusion, this study showed that PHD2 silencing significantly enhanced the protective paracrine effect of BM-MSCs on NEC, which was dependent on NF-κB mechanism. Future work should focus on the potential clinical use (e.g. optimizing the quality, dosing, timing and delivery strategy of CM) in order to develop innovative pharmacological agents suitable for NEC.

## Materials and methods

### Animals

Rat pups were delivered on day 21 of gestation by cesarean section under CO_2_ anesthesia from timed pregnant Sprague-Dawley rats provided by the Laboratory Animal Center of Sun Yat-Sen University (Guangzhou, China). Animals were used according to good animal practices, and animal experiments were approved by our local animal care and use committee. Animals were randomized by using a computerized random number generator to receive different treatments and investigators performing animal studies were blinded to the specific treatments.

### Cells

BM-MSCs were obtained, cultured and characterized based on our previously described procedures^8^. Nontransformed rat intestinal epithelial IEC-6 cells (CRL-1592, passage 13) was purchased from the American Type Culture Collection. IEC-6 cell lines were maintained in DMEM-H, supplemented with 10% fetal bovine serum, 1% Penicillin/Streptomycin in a 37 °C humidified incubator with 5% CO_2_. IEC-6 cells used in our experiments were at or before the 20th passage. All cells were tested for mycoplasma contamination (e-Myco plus, iNtRON Biotechnology, catalog number 25237) and determined to be negative.

### Experimental NEC model

NEC was induced using a well-established protocol based on gavage-feeding with hypercaloric formula, hypoxia, hypothermia, and oral administration of LPS^25,43^. No statistical methods were used to pre-determine sample size but our sample size was based on estimated effect size for similar settings in the previous studies^8,25^. Rats were either killed, and collected tissue samples on days 1, 4, and 7 for structural and functional examination, or were monitored for survival status throughout the 7-day course of the experiment. For in vitro studies, IEC-6 cells were treated with LPS (200 μg/mL) and H_2_O_2_ (0.2 mM) for 24 h and evaluated 1, 3, 5, and 7 days after exposure.

### Preparation and treatment of conditioned medium

For short hairpin RNA (shRNA) knockdown of PHD-2 in bone-marrow mesenchymal stem cells, the rat PHD2 (GenBank ID: XM_008772679) shRNA was constructed into the lentivirus gene transfer vector pGCSIL-GFP (Genechem Co., Ltd., Shanghai, China). BM-MSCs were infected with lentiviruses containing GFP or shPHD2-GFP at the MOI of 10. Stably transduced BM-MSCs were determined by detecting GFP fluorescence and PHD2 protein expression and enriched by cell sorting with FACS Aria II (BD Biosciences) for further culture and preparation of conditioned medium. The infection protocol of HIF-1α (GenBank ID: XM_006240196) shRNA was similar to that of lentiviral PHD2 shRNA. To prepare conditioned medium from BM-MSCs. BM-MSCs transduced with lentiviruses containing GFP (GFP-MSC) or shPHD2-GFP constructs (PHDMSC) was conditioning in new fresh serum-free DMEM-F12 for 48 h. In all, 1 mL of unconcentrated conditioned medium is approximately equivalent to a secretions of 2 × 10^5^ BM-MSCs. The conditioned medium was then collected and concentrated as MSC-CM and PHDMSC-CM, respectively. Conditioned medium was later concentrated 50-fold by ultrafiltration with a 5-kDa cut-off (Millipore, Billerica, MA) and stored at −20 °C for further use. Concentrated conditioned medium (First 0–3 days: 100 μl/animal/12 h) was used in experiments in vivo, whereas unconcentrated conditioned medium (2.5 ml/well/day) was used in experiments in vitro using six-well plate, unless otherwise specified.

To determine if IGF-1 or TGF-β2 substitutes for PHDMSC-CM in NEC model, pups (5–10 g) were injected 180 ng recombinant rat IGF-1 (ab52006, Abcam, Cambridge, UK) and 25 ng recombinant rat TGF-β2 (LS-G11803, LSBio, Seattle, USA) which is approximately equivalent to a IGF-1 or TGF-β2 content of 800 μl PHDMSC-CM in 8 doses delivered twice a day for 4 days.

For neutralizing experiments, the conditioned medium was collected and incubated for 30 min with control IgG or individual neutralizing antibodies for IGF-1 (1 μg/ml; Upstate Biotechnology), bFGF (0.5 μg/ml; R&D systems, Minneapolis, MN), VEGF (0.5 μg/ml; R&D systems, Minneapolis, MN), TGF-β1 (0.5 μg/ml; R&D systems, Minneapolis, MN), TGF-β2 (0.5 μg/ml; R&D systems, Minneapolis, MN), or combinations.

### Immunohistochemistry

Rats were euthanized and killed 1, 4, and 7 days after birth. The terminal ileum was harvested, fixed in 10% neutral-buffered formalin for 12 h, and then dehydrated and embedded with paraffin. Sections of 4 mm were used for H&E and other staining. Intestinal mucosal injury was graded by examining tissue sections with phase contrast microscopy using the grading scale described by Caplan et al.^44^. Intestinal morphologic changes were graded as: 0, normal tissue; 1, epithelial cell lifting; 2, necrosis to midvillus; 3, necrosis of entire villus; 4, transmural necrosis. Injury grades 2–4 were considered consistent with NEC. The number of positive cells in 5 crypts was scored in 100 crypts per section and reported as mean ± SD. Three rats were used in each group.

### Flow cytometry

Rabbit anti-rat CD29-FITC, CD34-FITC, CD44-FITC, and CD45-FITC (BD Bioscience, Franklin Lakes, NJ, USA) were used to identify the BM-MSCs phenotypes. For cell cycle detection and cell death, IEC-6 cells were analyzed by flow cytometry (BD Bioscience, Franklin Lakes, NJ, USA) based on our previously described procedures^8^. For intracellular staining, mesenteric lymph nodes were also carried out according to our previously described methods^8^.

### Cytokine array

Analysis of unconcentrated conditioned medium cytokines or blood samples cytokines were performed using the RayBio^®^ Biotin Label-based Rat Antibody Array 1 (RayBiotech, Norcross, GA, USA) according to the manufacturer’s instructions. To confirm the results, unconcentrated conditioned medium and serum samples was assayed by ELISA for selected cytokines.

### Gut permeability and gut motility

To evaluate the permeability of the intestine barrier, enzymatic-spectrophotometric method was used to detected plasma D-lactate concentrations. To evaluate intestinal motility in rats with or without NEC, methylene blue dye migration tests were carried out according to previously described methods^45^.

### Electron microscopy

Rats were euthanized and killed on day 4 of life. The intestinal specimens were fixed with 2.5% glutaraldehyde for 2 h before postfixation with 1% OsO4 and embedding in Epon 812. For contrasting, the air dried sections were stained with uranyl acetate. A Zeiss electron microscope (EM 902, Zeiss, Jena, Germany) was used for imaging.

### TUNEL staining

For in vivo studies, rats were euthanized and killed 1, 4, and 7 days after birth. Intestinal samples were collected for histopathological analysis of apoptosis by terminal deoxynucleotidyl transferase dUTP nick-end labeling (TUNEL) assay (In Situ Cell Death Detection Kit; Roche Applied Science, Indianapolis, IN). TUNEL-positive cells in 5 crypts was scored in 100 crypts per section and reported as mean ± SD. Three rats were used in each group. For in vitro studies, the number of TUNEL-positive cells detected in each field of view per well for four independent wells was reported as mean ± SD.

### Quantitative real-time PCR assay

Quantitative PCR was carried out using SYBR^®^ Premix ExTaq^TM^ (Takara) in the LightCycler 480 (Roche Applied Science,Indianapolis, IN). The real-time PCR primers of *Lgr5* is as follows: 5′-TGC CCT CCA ACC TCA GCG TCT T-3′(Forward), and 5′-AGG CCT GCG AAT GCT CCC TT-3′(Reverse). PCR cycles were 95 °C for 30 s, followed by 40 cycles of 95 °C for 10 s and 60 °C for 30 s. Reactions were performed in triplicate and analyzed individually, relative to β-actin (a normalization control), calculated using the 2^−ΔΔCt^ method. Thereafter, data for transcript expression levels were expressed as fold difference relative to negative control cells.

### Western blot

Rats were euthanized and killed at 1, 4, and 7 days after birth. Intestinal segments were prepared and carried out according to our previously described methods^8^. A β-actin antibody (Abcam, Cambridge, United Kingdom) at a 1:1000 dilution was used as the control.

### Nuclear protein extracts and EMSA

NF-κB was examined using electrophoretic mobility shift assay (EMSA). Nuclear extracts of BM-MSCs were prepared by hypotonic lysis followed by high salt extraction. EMSA was performed with the Gel Shift assay system (Promega, Madison, WI). In a typical experiment, The NF-κB consensus oligonucleotide probe (5′- AGT TGA GGG GAC TTT CCC AGG C -3′) (Promega) end-radiolabeled with [γ-32P]ATP (3000 Ci/mmol; Perkin-Elmer Life Technology, Waltham, MA) and T4 polynucleotide kinase (Promega) were incubated with nuclear extract, 100 µg/ml poly dI-dC, 10 mM Tris/HCl (pH 7.5), 50 mM NaCl, 0.5 mM EDTA, 0.5 mM DTT, 1 mM MgCl_2_, and 4% glycerol according to the manufacturer’s instructions. After the incubation, samples were charged on 4% native polyacrylamide gels with a 0.5 × TBE running buffer and visualized by autoradiography. A 100-fold molar excess of unlabeled competitor was added to the reaction mixture before adding the nuclear extracts in some experiments. BAY11-7082 (Sigma, 5 µM) and pCMV-IκBα-M, a dominant-negative form of IκBα (Clontech, Mountain View, CA) was used for inhibition of NF-κB activation.

### Chromatin immunoprecipitation assay

Four NF-κB binding sites in IGF-1 promoter and two NF-κB binding sites in TGF-β2 promoter were predicted with program ALGGEN-PROMO (http://alggen.lsi.upc.es/cgi-bin/promo_v3/promo/promoinit.cgi?dirDB=TF_8.3). ChIP assay for the IGF-1/TGF-β2 promoter region was performed using the EZ-ChIP kit (Millipore, Bedford, MA) according to the manufacturer’s instruction. Briefly, PHDMSC were cross-linked with 1% formaldehyde for 10 min at room temperature. To shear the chromatin, the whole cell extract was then sonicated after being resuspended in 0.45 ml lysis buffer. For IP, anti-NF-κB p65 and anti-NF-κB p50 (abcam, Cambridge, MA) were used. IP and Input DNA were purified using columns provided in the EZ-ChIP kit and then amplified. PCR was performed to amplify rat IGF-1 and TGF-β2 promoter fragments using specific primer pairs (Supplementary Table 1). All ChIP assays were repeated three or more times.

### Statistical analysis

Data were analyzed using SPSS 17.0 software (SPSS Inc., Chicago, IL, USA) and expressed as mean ± SD for normally distributed data examined by the Shapiro-Wilk method. Student’s *t*-test or one or two-way ANOVA with the Bonferroni post-hoc test were done to determine statistical significance. Animal survival curves were analyzed using the Kaplan-Meier method. Statistical values of *P* < 0.05 were considered to be significant.

## Supplementary information

Supplemental legends

Supplemental table

Supplementary Figure 1

Supplementary Figure 2

Supplementary Figure 3

Supplementary Figure 4
