# Supplementary material for: Prolyl hydroxylase 2 silencing enhances the paracrine effects of mesenchymal stem cells on necrotizing enterocolitis in an NF-κB-dependent mechanism
Source: Cell Death Dis. 2020 Mar 16;11(3):188. doi: 10.1038/s41419-020-2378-3 (PMC7075868; doi:10.1038/s41419-020-2378-3)
Supplement: Supplementary file 1 — Supplemental legends [file 41419_2020_2378_MOESM1_ESM.doc]

**Supplementary Figure Legends**

Supplementary Figure 1. Comparison of survival in NEC rats between sexes. (A) Cumulative survival for all NEC rats with different sexes was analyzed using the Kaplan-Meier method. In all NEC rat pups, there is no significant difference on survival between females and males (39.28% vs. 37.14%). (B) Cumulative survival for NEC rats in PHDMSC-CM group with different sexes was analyzed using the Kaplan-Meier method. The improvement of survival after PHDMSC-CM was virtually identical in both sexes: by 40.1% in females (23.53% without PHDMSC-CM vs. 63.63% with PHDMSC-CM) and by 35.2% in males (21.05 vs. 56.25%). The cumulated number of rats in each experimental group is presented in parenthesis. *P*-values were determined by log-rank testing.

Supplementary Figure 2. Changes of the IGF-1 and TGF-β2 contents in the intestinal mucosa. **, *P* < 0.05 versus NEC + DMEM-F12. ##, *P* < 0.05 versus NEC + MSC-CM.

Supplementary Figure 3. CM from LPS-treated MSCs provides beneficial effects on apoptosis and proliferation of LPS/H_2_O_2_ treated IEC-6 cell. BM-MSCs were stimulated with NF-κB stimulator LPS (1 μg/ml for 30min). The apoptosis and proliferation were evaluated by quantification of TUNEL-positive and PCNA-positive IEC-6 cells *in vitro* 3 days after exposure, respectively. N=6. **, *P* < 0.05 versus NEC + PHDMSC-CM. ##, *P* < 0.05 versus NEC+DMEM-F12.

Supplementary Figure 4. Quantification of IGF-1 and TGF-β2 mRNA expression by qPCR in BM-MSCs treated with NF-κB stimulator lipopolysaccharide (LPS, 1 μg/ml) at indicated time points. These results are representative of 3 independent experiments. **, *P* < 0.05 versus LPS (0 minutes).
