# Supplementary material for: Prolyl hydroxylase 2 silencing enhances the paracrine effects of mesenchymal stem cells on necrotizing enterocolitis in an NF-κB-dependent mechanism
Source: Cell Death Dis. 2020 Mar 16;11(3):188. doi: 10.1038/s41419-020-2378-3 (PMC7075868; doi:10.1038/s41419-020-2378-3)
Supplement: Supplementary file 2 — Supplemental table [file 41419_2020_2378_MOESM2_ESM.doc]

**Supplementary Tables:**

**Table S1. Primers used for ChIP assays (rat genes)**

Primer sequence

Primer name

Forward (5’ to 3’) Reverse (5’ to 3’)

For ChIP assays

*rIGF-1*-a 5’-ACTGTATGAGCAGGGATTT 5’-AGAGAATGCTATGTGAGAGG

*rIGF-1*-b 5’-ATGTGTCAGTACCTTCAAATC 5’-GCGATAGATAGGAAAGTGAA

*rIGF-1*-c 5’-CCGTCTATAGGTTATAGGAAA 5’-GACTATCTCCAGAAAGCACA

*rTGF-β1*-a 5’-CCTATGTGCACAACAGATTT 5’-TGTACAGAGAGGACACAGAGT

*rTGF-β1*-b 5’-TACTGATCCTGAGGGGTACT 5’-CACATTGGAATACAGTGGTT
