# Supplementary figures and images for: Prolyl hydroxylase 2 silencing enhances the paracrine effects of mesenchymal stem cells on necrotizing enterocolitis in an NF-κB-dependent mechanism
Source: Cell Death Dis. 2020 Mar 16;11(3):188. doi: 10.1038/s41419-020-2378-3 (PMC7075868; doi:10.1038/s41419-020-2378-3)

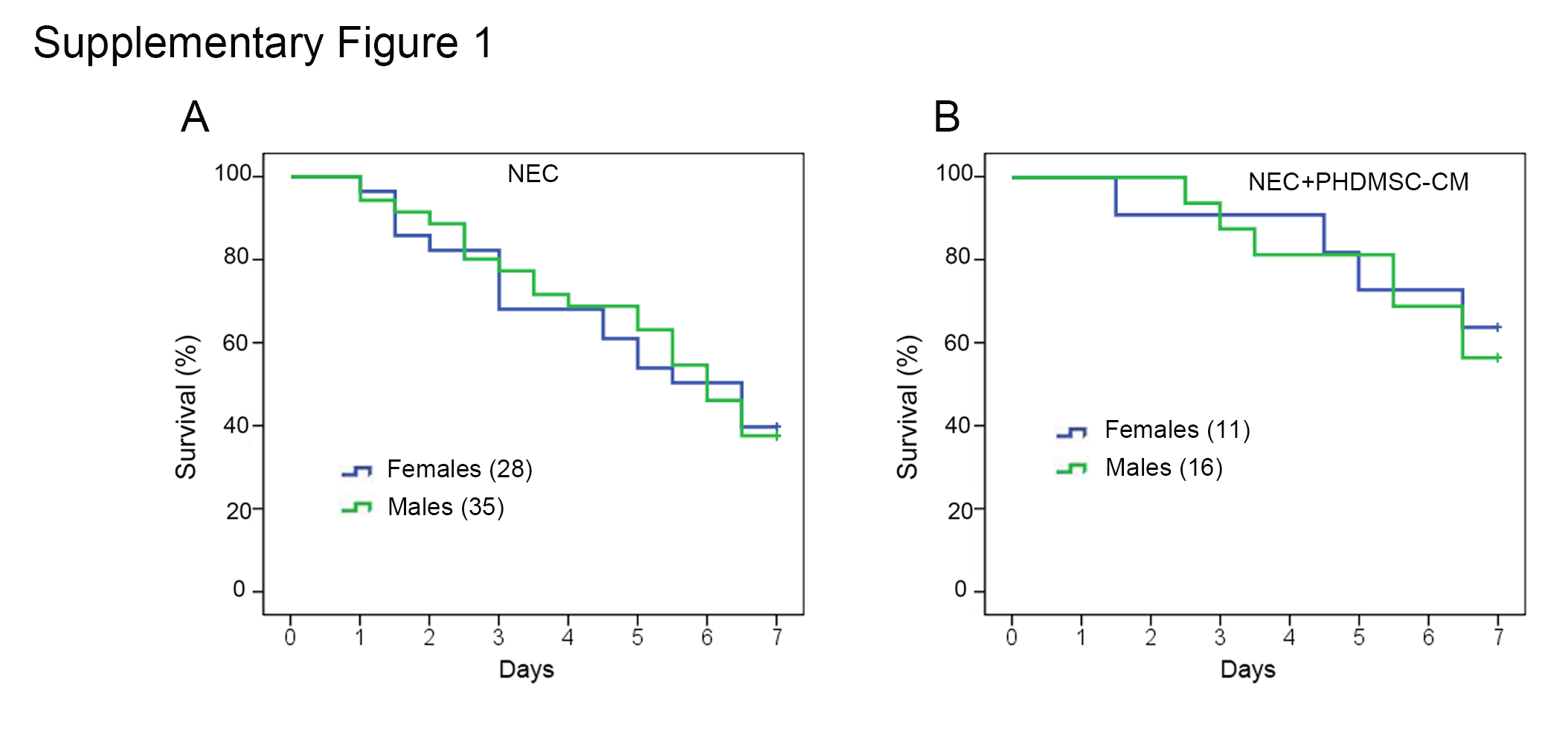

Supplement: Supplementary file 3 — Supplementary Figure 1 [file 41419_2020_2378_MOESM3_ESM.tif]

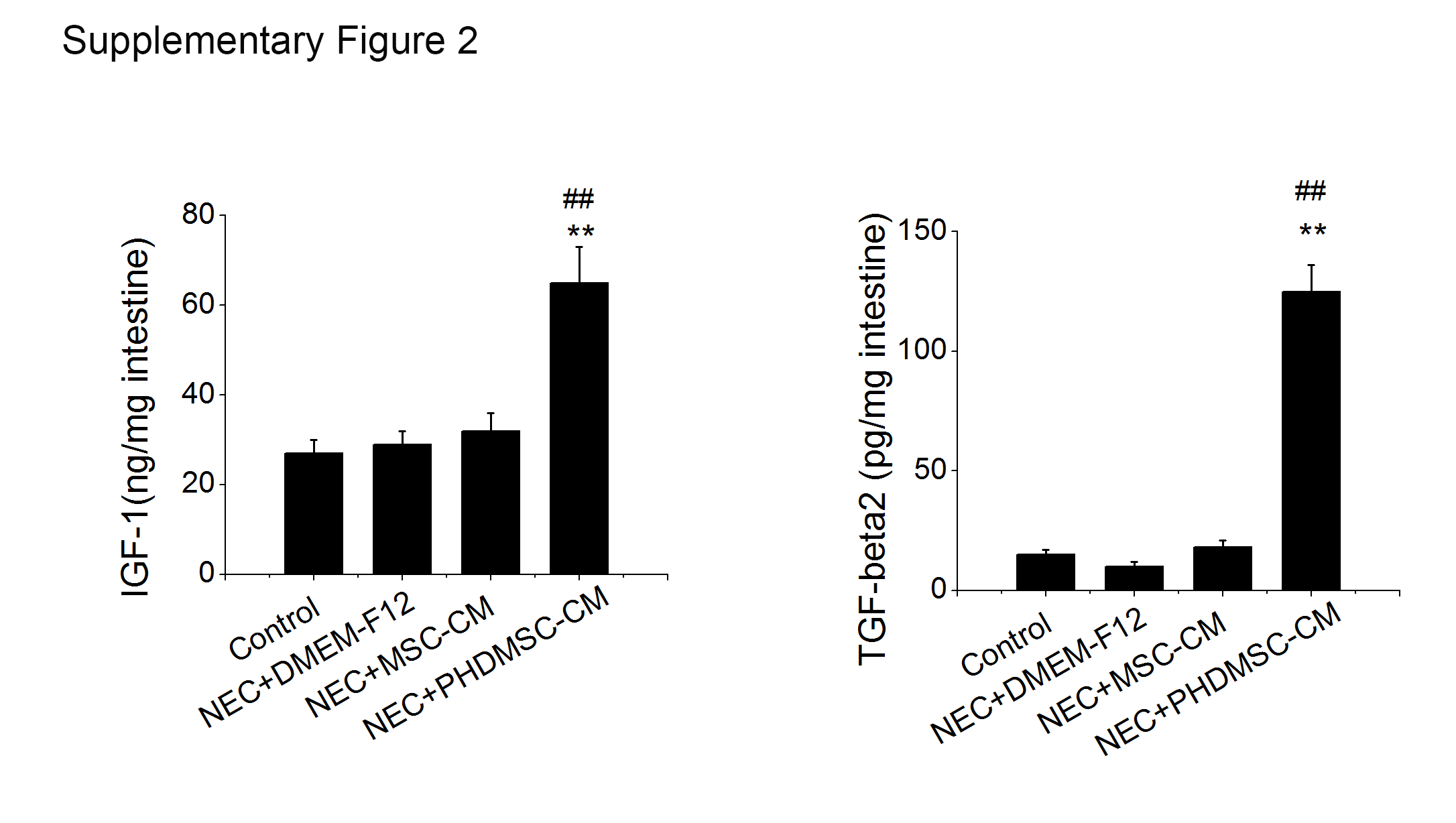

Supplement: Supplementary file 4 — Supplementary Figure 2 [file 41419_2020_2378_MOESM4_ESM.tif]

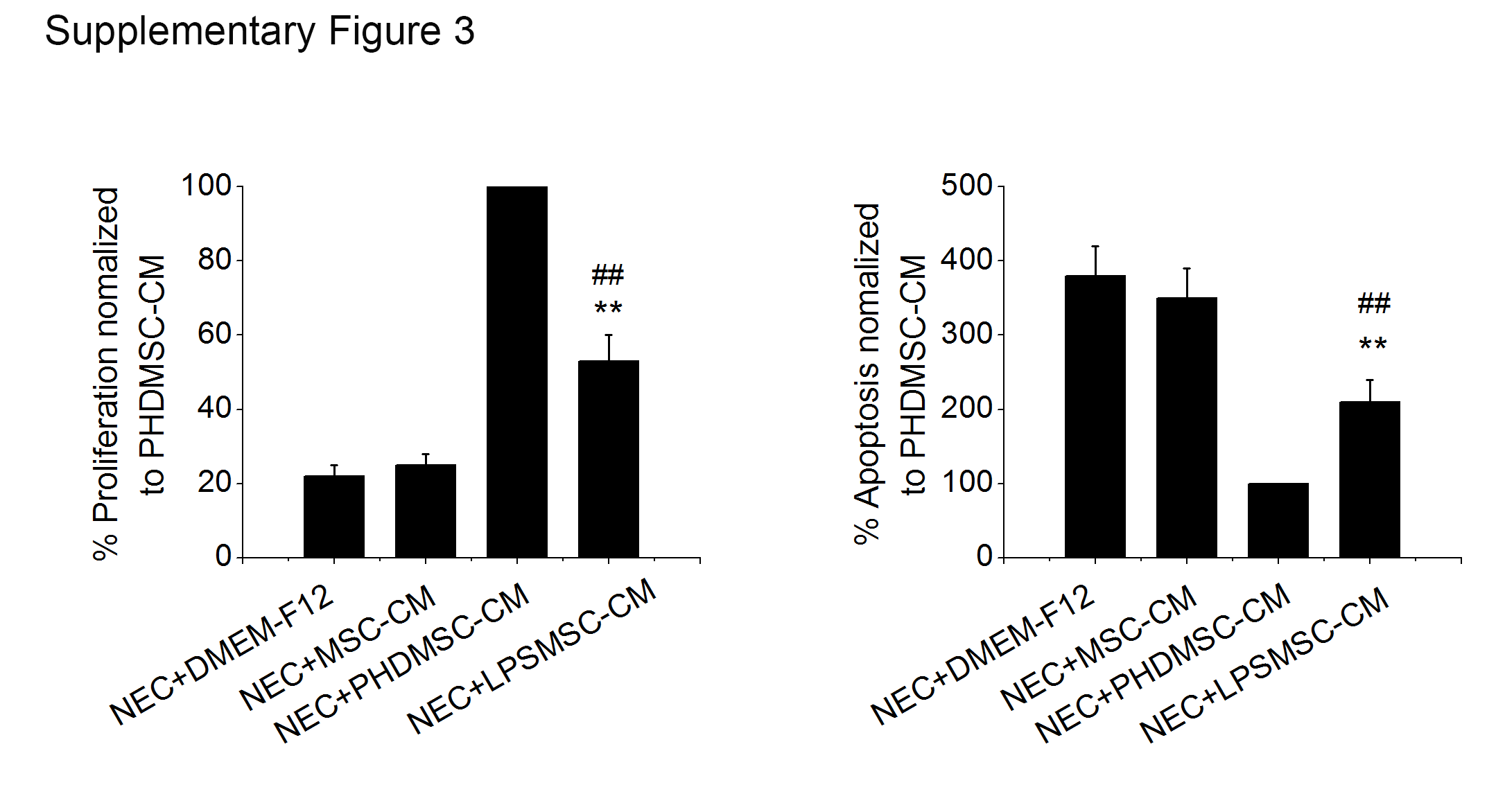

Supplement: Supplementary file 5 — Supplementary Figure 3 [file 41419_2020_2378_MOESM5_ESM.tif]

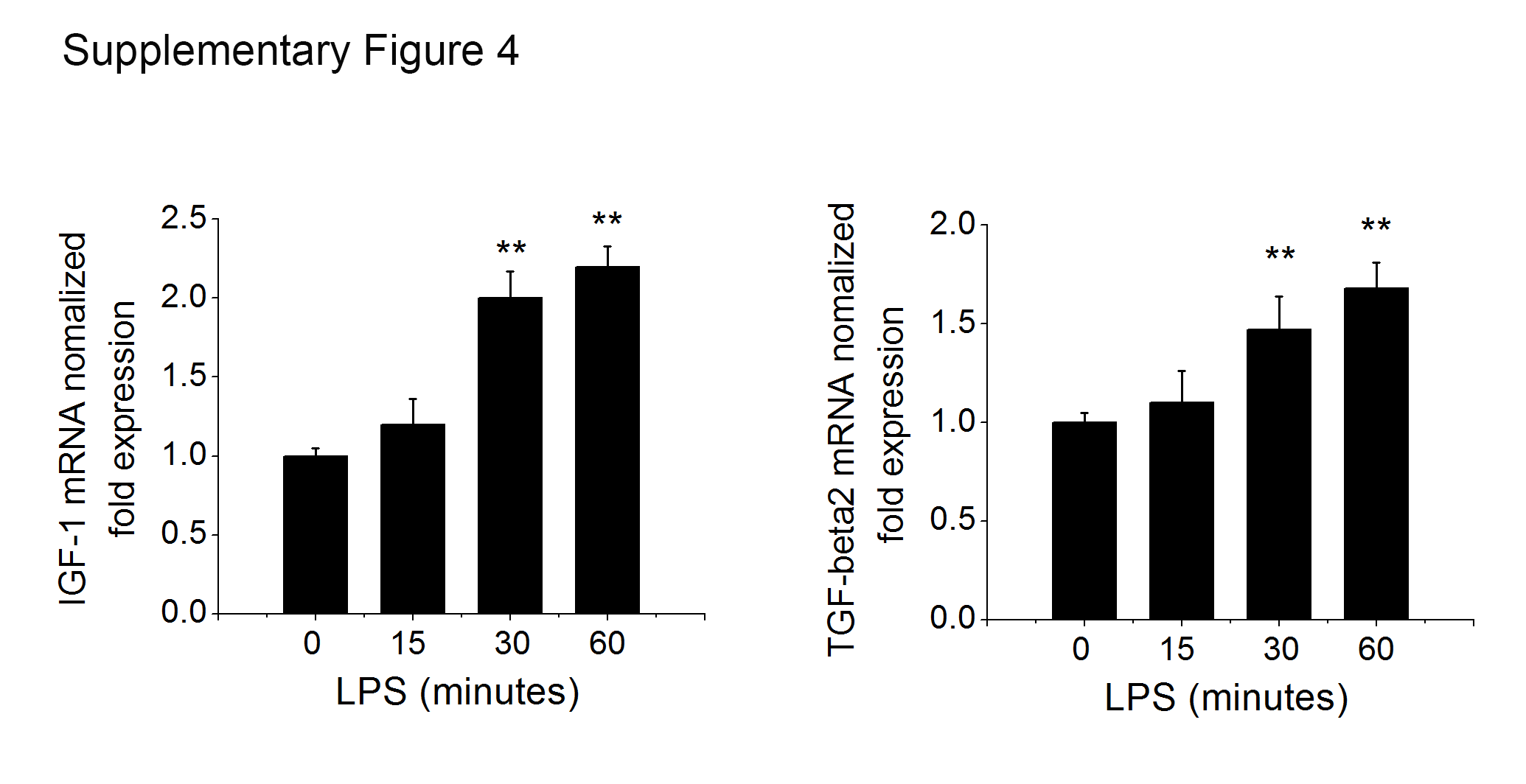

Supplement: Supplementary file 6 — Supplementary Figure 4 [file 41419_2020_2378_MOESM6_ESM.tif]
